# A new test of multivariate nonlinear causality

**DOI:** 10.1371/journal.pone.0185155

**Published:** 2018-01-05

**Authors:** Zhidong Bai, Yongchang Hui, Dandan Jiang, Zhihui Lv, Wing-Keung Wong, Shurong Zheng

**Affiliations:** 1 KLASMOE and School of Mathematics and Statistics, Northeast Normal University, Changchun, China; 2 School of Mathematics and Statistics, Xi’an Jiaotong University, Xi’an, China; 3 School of Mathematics, Jilin University, Changchun, China; 4 Department of Finance, Asia University, Taichung, Taiwan; 5 Department of Economics, Lingnan University, Hong Kong, China; Feng Chia University, TAIWAN

## Abstract

The multivariate nonlinear Granger causality developed by Bai et al. (2010) (Mathematics and Computers in simulation. 2010; 81: 5-17) plays an important role in detecting the dynamic interrelationships between two groups of variables. Following the idea of Hiemstra-Jones (HJ) test proposed by Hiemstra and Jones (1994) (Journal of Finance. 1994; 49(5): 1639-1664), they attempt to establish a central limit theorem (CLT) of their test statistic by applying the asymptotical property of multivariate *U*-statistic. However, Bai et al. (2016) (2016; arXiv: 1701.03992) revisit the HJ test and find that the test statistic given by HJ is NOT a function of *U*-statistics which implies that the CLT neither proposed by Hiemstra and Jones (1994) nor the one extended by Bai et al. (2010) is valid for statistical inference. In this paper, we re-estimate the probabilities and reestablish the CLT of the new test statistic. Numerical simulation shows that our new estimates are consistent and our new test performs decent size and power.

## Introduction

After the pioneering work of Granger [1], Granger causality tests have been developed into a set of useful methods to detect causal relations between time series in economics and finance. Linear Granger causality tests within the linear autoregressive model class have been developed in many directions, e.g., Hurlin et al. [2] proposed a procedure for causality tests with panel data, Ghysels et al. [3] test for Granger causality with mixed frequency data based on the multiple-horizon framework established by Dufour and Renault [4] and Dufour et al. [5]. Though linear tests of Granger causality have been investigated very deeply, they are limited in their capability to detect nonlinear causality.

The real world is “almost certainly nonlinear” as Granger [6] notes, so it is more important to test the nonlinear causality. Baek and Brock [7] develop a nonlinear Granger causality test which is modified by Hiemstra and Jones [8] later on to study the bivariate nonlinear causal relationship between two series. Among the various tests of nonlinear Granger causality, the Hiemstra-Jones test (hereafter, the HJ test) proposed by Hiemstra and Jones [8] is the most cited by scholars and the most frequently applied by practitioners in economics and finance. There were over 1100 Google Scholar hits by September 2016, which illustrates its significance in the economics and finance literatures.

Bai et al. [9] extend the HJ test from bivariate setting to multivariate setting catering to the practical needs that economic and financial factors usually move together and influence others in groups. This extension encourages a large amount of applications. For example, Lam et al. [10] suggest to use such technics to make better investment decisions. Zheng and Chen [11] proposed a complete double selection method in identifying external influential factors for a particular stock market. Choudhry et al. [12] investigates the nonlinear dynamic co-movements between gold returns, stock market returns and stock market volatility during the recent global financial crisis. Choudhry et al. [13] investigate the relationship between stock market volatility and the business cycle in four major economies US, Canada, Japan and the UK.

However, several works note that counterintuitive results are obtained from the HJ test, Diks and Panchenko ([14, 15]) find that the HJ test is seriously over-rejecting in simulation studies. In accordance with the evidence presented by Diks and Panchenko ([14, 15]), Bai et al. [16] reinvestigate the HJ test and reveal some of the underlying reasons for the questionable performance of HJ test. They find that the estimators of the probabilities in the definition are not *U*-statistics as Hiemstra and Jones [8] claimed and the central limit theorem of the test statistics is not valid. Bai et al. [16] propose a set of consistent estimators of the probabilities in the definition of Hiemstra and Jones [8] and provide a new test statistic with its asymptotic distribution.

Considering the significant importance of the multivariate nonlinear Granger causality test, there is an urgent need to reinvestigate Bai et al. [9] and extend Bai et al. [16] to multivariate setting. The remainder of this paper is organized as follows. In Section 2, we simply review the procedure of the multivariate nonlinear Granger causality test (here after BWZ test) extended by Bai et al. [9]. In Section 3, we re-estimate the probabilities in the definition of Bai et al. [9] and establish the asymptotic distribution of the new test statistics. Simulation results are presented in Section 4. Finally, we provide some concluding remarks in Section 5.

## The multivariate nonlinear causality test extended from HJ test

Bai et al. [9] consider two strictly stationary and weakly dependent vector time series processes *X*_*t*_ = (*X*_1,*t*_, *X*_2,*t*_, ⋯, *X*_*n*_1_,*t*_)′, *Y*_*t*_ = (*Y*_1,*t*_, *Y*_2,*t*_, ⋯, *Y*_*n*_2_,*t*_)′. The *m*_*x*_*i*__-length lead vector of *X*_*i*,*t*_ is defined as $X_{i,t}^{m_{x_{i}}}\equiv \left(X_{i,t},X_{i,t+1},\cdots ,X_{i,t+m_{x_{i}}-1}\right),m_{x_{i}}=1,2,\cdots ,t=1,2,\cdots ,$ similarly *L*_*x*_*i*__-length lag vector of *X*_*i*,*t*_, and *L*_*y*_*i*__-length lag vector of *Y*_*i*,*t*_ are defined as $X_{i,t-L_{x_{i}}}^{L_{x_{i}}}\equiv \left(X_{i,t-L_{x_{i}}},X_{i,t-L_{x_{i}}+1},\cdots ,X_{i,t-1}\right),L_{x_{i}}=1,2,\cdots ,t=L_{x_{i}}+1,L_{x_{i}}+2,\cdots ,Y_{i,t-L_{y_{i}}}^{L_{y_{i}}}\equiv \left(Y_{i,t-L_{y_{i}}},Y_{i,t-L_{y_{i}}+1},\cdots ,Y_{i,t-1}\right),L_{y_{i}}=1,2,\cdots ,t=L_{y_{i}}+1,L_{y_{i}}+2,\cdots$. Denote *M*_*x*_ = (*m*_*x*_1__, ⋯, *m*_*x*_*n*_1___), *m*_*x*_ = max(*m*_*x*_1__, ⋯, *m*_*x*_*n*_1___), *L*_*x*_ = (*L*_*x*_1__, ⋯, *L*_*x*_*n*_1___), *l*_*x*_ = max(*L*_*x*_1__, ⋯, *L*_*x*_*n*_1___), *L*_*y*_ = (*L*_*y*_1__, ⋯, *L*_*y*_*n*_2___), *l*_*y*_ = max(*L*_*y*_1__, ⋯, *L*_*y*_*n*_2___). For given *M*_*x*_, *L*_*x*_, *L*_*y*_, *e*, Bai et al. [9] define that

$$\begin{array}{cc} \{∥X_{t}^{M_{x}}-X_{s}^{M_{x}}∥<e\}\equiv & \left\{∥X_{i,t}^{m_{x_{i}}}-X_{i,s}^{m_{x_{i}}}∥<e,\;for\;all\;i=1,2,\cdots ,n_{1}\right\}\\ \{∥X_{t-L_{x}}^{L_{x}}-X_{s-L_{x}}^{L_{x}}∥<e\}\equiv & \left\{∥X_{i,t-L_{x_{i}}}^{L_{x_{i}}}-X_{i,s-L_{x_{i}}}^{L_{x_{i}}}∥<e,\;for\;all\;i=1,2,\cdots ,n_{1}\right\}\\ \{∥Y_{t-L_{y}}^{L_{y}}-Y_{s-L_{y}}^{L_{y}}∥<e\}\equiv & \{∥Y_{i,t-L_{y_{i}}}^{L_{y_{i}}}-Y_{i,s-L_{y_{i}}}^{L_{y_{i}}}∥<e,\;for\;all\;i=1,2,\cdots ,n_{2}\}\;, \end{array}$$

where ‖⋅‖ denotes the maximum norm defined as ‖*X* − *Y*‖ = max(|*x*_1_ − *y*_1_|, |*x*_2_ − *y*_2_|, ⋯, |*x*_*n*_ − *y*_*n*_|) for any two vectors *X* = (*x*_1_, ⋯, *x*_*n*_) and *Y* = (*y*_1_, ⋯, *y*_*n*_).

**Definition 1** *The vector time series* {*Y*_*t*_} *does not strictly Granger cause another vector time series* {*X*_*t*_} *if*

$$\begin{array}{ccc} & & P(∥X_{t}^{M_{x}}-X_{s}^{M_{x}}∥<e|∥X_{t-L_{x}}^{L_{x}}-X_{s-L_{x}}^{L_{x}}∥<e,∥Y_{t-L_{y}}^{L_{y}}-Y_{s-L_{y}}^{L_{y}}∥<e)\\ & & \;=P(∥X_{t}^{M_{x}}-X_{s}^{M_{x}}∥<e|∥X_{t-L_{x}}^{L_{x}}-X_{s-L_{x}}^{L_{x}}∥<e)\;, \end{array}$$ (1)

*where* *P*(⋅|⋅) *denotes conditional probability*.

Using the notation

$$\begin{array}{ccc} C_{1}\left(M_{x}+L_{x},L_{y},e\right) & \equiv & P(∥X_{t-L_{x}}^{M_{x}+L_{x}}-X_{s-L_{x}}^{M_{x}+L_{x}}∥<e,∥Y_{t-L_{y}}^{L_{y}}-Y_{s-L_{y}}^{L_{y}}∥<e)\\ C_{2}\left(L_{x},L_{y},e\right) & \equiv & P(∥X_{t-L_{x}}^{L_{x}}-X_{s-L_{x}}^{L_{x}}∥<e,∥Y_{t-L_{y}}^{L_{y}}-Y_{s-L_{y}}^{L_{y}}∥<e)\\ C_{3}\left(M_{x}+L_{x},e\right) & \equiv & P(∥X_{t-L_{x}}^{M_{x}+L_{x}}-X_{s-L_{x}}^{M_{x}+L_{x}}∥<e)\\ C_{4}\left(L_{x},e\right) & \equiv & P(∥X_{t-L_{x}}^{L_{x}}-X_{s-L_{x}}^{L_{x}}∥<e)\;, \end{array}$$

Bai, et al. [9] re-express Eq (1) as

$$\begin{array}{c} \frac{C_{1}\left(M_{x}+L_{x},L_{y},e\right)}{C_{2}\left(L_{x},L_{y},e\right)}=\frac{C_{3}\left(M_{x}+L_{x},e\right)}{C_{4}\left(L_{x},e\right)}\;. \end{array}$$ (2)

For two sets of simultaneous samples {*x*_*i*,*t*_, *i* = 1, ⋯, *n*_1_, *t* = 1, ⋯, *T*} and {*y*_*i*,*t*_, *i* = 1, ⋯, *n*_2_, *t* = 1, ⋯, *T*}, they propose the following test statistic

$$\begin{array}{c} \sqrt{n}\left(\frac{C_{1}\left(M_{x}+L_{x},L_{y},e,n\right)}{C_{2}\left(L_{x},L_{y},e,n\right)}-\frac{C_{3}\left(M_{x}+L_{x},e,n\right)}{C_{4}\left(L_{x},e,n\right)}\right)\;, \end{array}$$ (3)

where

$$\begin{array}{ccc} & & C_{1}\left(M_{x}+L_{x},L_{y},e,n\right)\\ & & \;\equiv \frac{2}{n(n-1)}\underset{t<s}{\sum \sum }\prod_{i=1}^{n_{1}}I\left(x_{i,t-L_{x_{i}}}^{m_{x_{i}}+L_{x_{i}}},x_{i,s-L_{x_{i}}}^{m_{x_{i}}+L_{x_{i}}},e\right)\cdot \prod_{i=1}^{n_{2}}I\left(y_{i,t-L_{y_{i}}}^{L_{y_{i}}},y_{i,s-L_{y_{i}}}^{L_{y_{i}}},e\right),\\ & & \;C_{2}\left(L_{x},L_{y},e,n\right)\equiv \frac{2}{n(n-1)}\underset{t<s}{\sum \sum }\prod_{i=1}^{n_{1}}I\left(x_{i,t-L_{x_{i}}}^{L_{x_{i}}},x_{i,s-L_{x_{i}}}^{L_{x_{i}}},e\right)\cdot \prod_{i=1}^{n_{2}}I\left(y_{i,t-L_{y_{i}}}^{L_{y_{i}}},y_{i,s-L_{y_{i}}}^{L_{y_{i}}},e\right),\\ & & \;C_{3}\left(M_{x}+L_{x},e,n\right)\equiv \frac{2}{n(n-1)}\underset{t<s}{\sum \sum }\prod_{i=1}^{n_{1}}I\left(x_{i,t-L_{x_{i}}}^{m_{x_{i}}+L_{x_{i}}},x_{i,s-L_{x_{i}}}^{m_{x_{i}}+L_{x_{i}}},e\right),\\ & & \;C_{4}\left(L_{x},e,n\right)\equiv \frac{2}{n(n-1)}\underset{t<s}{\sum \sum }\prod_{i=1}^{n_{1}}I\left(x_{i,t-L_{x_{i}}}^{L_{x_{i}}},x_{i,s-L_{x_{i}}}^{L_{x_{i}}},e\right), \end{array}$$

and

$$\begin{array}{c} I\left(x,y,e\right)=\left\{ \begin{array}{cc} 0, & \text{if}∥x-y∥>e\\ 1, & \text{if}\;∥x-y∥\leq e \end{array}\right.. \end{array}$$

**Remark:** Following the instruction of Hiemstra and Jones [8], Bai et al. [9] take *C*_*j*_(∗, *n*)s as multivariate *U*-statistic estimators of their counterparts *C*_*j*_(∗)s and apply the asymptotic property of *U*-statistic to show the limiting results for the test statistics (3). However the *C*_*j*_(∗, *n*)s are not *U*-statistics, because the expectations of the general terms are not the same. Moreover, the *C*_*j*_(∗)s are related to the indices *t* and *s* (in fact, related to |*t* − *s*| for strongly stationary processes), while the *C*_*j*_(∗, *n*)s were independent of *t* and *s* for summing up over them. Therefore, the *C*_*j*_(∗, *n*) estimators are neither consistent nor asymptotic normal estimators of their counterparts *C*_*j*_(∗).

## A new multivariate nonlinear causality test

We first remind the reader that the pair (*s*, *t*) (in fact, |*t* − *s*| for strongly stationary processes) in Eq (1) of Definition 1 is a key parameter of the probabilities *C*_*j*_(∗). In fact, both Hiemstra and Jones [8] and Bai et al. [9] note this, and there is no problem in Eq (1) of Definition 1. However, it seems that they overlooked this fact in their proposed estimation of *C*_*j*_(∗). The improper estimators *C*_*j*_(∗, *n*) thus lead to an invalid asymptotic distribution of the test statistic.

We now begin to state the procedure for our new test. If {*X*_*i*,1_} and {*Y*_*j*,1_}, *i* ∈ {1, 2, ⋯, *n*_1_}, *j* ∈ {1, 2, ⋯, *n*_2_} share a common standard deviation 1, and thereby share a common scale parameter. For any given pair (*s*, *t*), we denote

$$\begin{array}{ccc} & & C_{1}\left(M_{x}+L_{x},L_{y},e;t,s\right)\equiv P\left(∥X_{t-L_{x}}^{M_{x}+L_{x}}-X_{s-L_{x}}^{M_{x}+L_{x}}∥<e,∥Y_{t-L_{y}}^{L_{y}}-Y_{s-L_{y}}^{L_{y}}∥<e\right)\\ & & C_{2}\left(L_{x},L_{y},e;t,s\right)\equiv P\left(∥X_{t-L_{x}}^{L_{x}}-X_{s-L_{x}}^{L_{x}}∥<e,∥Y_{t-L_{y}}^{L_{y}}-Y_{s-L_{y}}^{L_{y}}∥<e\right)\\ & & C_{3}\left(M_{x}+L_{x},e;t,s\right)\equiv P\left(∥X_{t-L_{x}}^{M_{x}+L_{x}}-X_{s-L_{x}}^{M_{x}+L_{x}}∥<e\right)\\ & & C_{4}\left(L_{x},e;t,s\right)\equiv P\left(∥X_{t-L_{x}}^{L_{x}}-X_{s-L_{x}}^{L_{x}}∥<e\right) \end{array}$$

Under the assumption of the stationary, for the given pair (*s*, *t*), if |*t* − *s*| = *l*, we denote *C*_1_(*M*_*x*_ + *L*_*x*_, *L*_*y*_, *e*; *t*, *s*) ≡ *C*_1_(*M*_*x*_ + *L*_*x*_, *L*_*y*_, *e*; *t*, *l*), which does not depend on t, so we can write *C*_1_(*M*_*x*_ + *L*_*x*_, *L*_*y*_, *e*; *l*) instead of *C*_1_(*M*_*x*_ + *L*_*x*_, *L*_*y*_, *e*, *t*; *l*), the same to the others. So under the assumption of strictly stationary, for each *l* > 0, we examine whether there is nonlinear Granger causality from {*Y*_*t*_} to {*X*_*t*_} by testing the following hypothesis

$$\begin{array}{c} H_{0}\;:\;\;\frac{C_{1}\left(M_{x}+L_{x},L_{y},e;l\right)}{C_{2}\left(L_{x},L_{y},e;l\right)}=\frac{C_{3}\left(M_{x}+L_{x},e;l\right)}{C_{4}\left(L_{x},e;l\right)}\;. \end{array}$$ (4)

If we consider two sets of simultaneous samples (To implement the test, each series is standardized at first so that all series share a common standard deviation, and thereby share a common scale parameter.) {*x*_*i*,*t*_, *i* = 1, ⋯, *n*_1_, *t* = 1, ⋯, *T*} and {*y*_*j*,*t*_, *j* = 1, ⋯, *n*_2_, *t* = 1, ⋯, *T*}, we first provide the consistent estimators of *C*_1_(*M*_*x*_ + *L*_*x*_, *L*_*y*_, *e*; *l*), *C*_2_(*L*_*x*_, *L*_*y*_, *e*; *l*), *C*_3_(*M*_*x*_ + *L*_*x*_, *e*; *l*) and *C*_4_(*L*_*x*_, *e*; *l*) are

$$\begin{array}{ccc} & & {\hat{C}}_{1}\left(M_{x}+L_{x},L_{y},e;l\right)\equiv \frac{1}{n}\sum_{t=L_{xy}+1}^{T-l-m_{x}+1}\prod_{i=1}^{n_{1}}I\left(x_{i,t-L_{x_{i}}}^{m_{x_{i}}+L_{x_{i}}},x_{i,t+l-L_{x_{i}}}^{m_{x_{i}}+L_{x_{i}}},e\right)\cdot \prod_{j=1}^{n_{2}}I\left(y_{j,t-L_{y_{j}}}^{L_{y_{j}}},y_{j,t+l-L_{y_{j}}}^{L_{y_{j}}},e\right),\\ & & {\hat{C}}_{2}\left(L_{x},L_{y},e;l\right)\equiv \frac{1}{n}\sum_{t=L_{xy}+1}^{T-l-m_{x}+1}\prod_{i=1}^{n_{1}}I\left(x_{i,t-L_{x_{i}}}^{L_{x_{i}}},x_{i,t+l-L_{x_{i}}}^{L_{x_{i}}},e\right)\cdot \prod_{j=1}^{n_{2}}I\left(y_{j,t-L_{y_{j}}}^{L_{y_{j}}},y_{j,t+l-L_{y_{j}}}^{L_{y_{j}}},e\right),\\ & & {\hat{C}}_{3}\left(M_{x}+L_{y},e;l\right)\equiv \frac{1}{n}\sum_{t=L_{xy}+1}^{T-l-m_{x}+1}\prod_{i=1}^{n_{1}}I\left(x_{i,t-L_{x_{i}}}^{m_{x_{i}}+L_{x_{i}}},x_{i,t+l-L_{x_{i}}}^{m_{x_{i}}+L_{x_{i}}},e\right),\\ & & {\hat{C}}_{4}\left(L_{x},e;l\right)\equiv \frac{1}{n}\sum_{t=L_{xy}+1}^{T-l-m_{x}+1}\prod_{i=1}^{n_{1}}I\left(x_{i,t-L_{x_{i}}}^{L_{x_{i}}},x_{i,t+l-L_{x_{i}}}^{L_{x_{i}}},e\right), \end{array}$$

where *L*_*xy*_ = max(*L*_*x*_, *L*_*y*_), *n* = *T* − *L*_*xy*_ − *l* − *m*_*x*_ + 1.

The consistency of our proposed estimators can be shown straightforwardly and the detail of the proof is omitted. We use a simple numerical study to show that our estimators are consistent whereas those of Bai et al. [9] are not. Let

$$\begin{array}{c} \left(\begin{array}{c} X_{1,t}\\ X_{2,t} \end{array}\right)=\left(\begin{array}{cc} 1 & 0\\ 0 & 1 \end{array}\right)\left(\begin{array}{c} a_{1,t-1}\\ a_{2,t-1} \end{array}\right)+\left(\begin{array}{c} a_{1,t}\\ a_{2,t} \end{array}\right), \end{array}$$

where {*a*_1,*t*_}, {*a*_2,*t*_} are i.i.d. and mutually independent random variables generated from *N*(0, 1), while {*Y*_*t*_} could be any stationary sequence. Let *l* = 1, *L*_*x*_ = *L*_*y*_ = *M*_*x*_ = 1. We can calculate the exact values of *C*_4_(*L*_*x*_, *e*; *l*), which are 0.2709 and 0.5057, respectively, when *e* = 1 and *e* = 1.5. For simplicity, we denote the true value of *C*_4_(*L*_*x*_, *e*; *l*), the estimate proposed by Bai et al. [9] and our new estimate as *C*_4_, ${\hat{C}}_{4}^{BWZ}$ and ${\hat{C}}_{4}$, respectively, in Table 1. Additionally, Table 1 provides the estimated values with their corresponding relative estimation errors in brackets when *T* = 1000, 2000 and 4000. It is obvious that ${\hat{C}}_{4}^{BWZ}$ is not consistent.

**Table 1:** *C*_4_(*L*_*x*_, *e*; *l*) and estimated values.

*T* =	*e* = 1			*e* = 1.5		
	*C*_4_	${\hat{C}}_{4}$	${\hat{C}}_{4}^{BWZ}$	*C*_4_	${\hat{C}}_{4}$	${\hat{C}}_{4}^{BWZ}$
1000	0.2709	0.2497(7.83%)	0.0154(94.32%)	0.5057	0.4774(5.60%)	0.0732(85.53%)
2000	0.2709	0.2639(2.58%)	0.0176(93.50%)	0.5057	0.4847(4.15%)	0.0795(84.28%)
4000	0.2709	0.2692(0.63%)	0.0193(92.88%)	0.5057	0.4909(2.93%)	0.0820 (83.78%)

Note: The true value of *C*_4_(*L*_*x*_, *e*; *l*) is denoted *C*_4_, the BWZ estimate and our new estimate are denoted ${\hat{C}}_{4}^{BWZ}$ and ${\hat{C}}_{4}$, respectively. The relative estimation errors are in the accompanying brackets.

Now, we propose

$$\begin{array}{c} T_{n}=\sqrt{n}\left(\frac{{\hat{C}}_{1}\left(M_{x}+L_{x},L_{y},e,l\right)}{{\hat{C}}_{2}\left(L_{x},L_{y},e,l\right)}-\frac{{\hat{C}}_{3}\left(M_{x}+L_{x},e,l\right)}{{\hat{C}}_{4}\left(L_{x},e,l\right)}\right) \end{array}$$ (5)

as the test statistic, and we derive the following asymptotic distribution of *T*_*n*_ for the Granger causality test.

**Theorem 1** *Stationary sequences* {*x*_*i*,*t*_, *i* = 1, ⋯, *n*_1_, *t* = 1, ⋯, *T*} *and* {*y*_*j*,*t*_, *j* = 1, ⋯, *n*_2_, *t* = 1, ⋯, *T*} *are strong mixing, with mixing coefficients satisfying the conditions of Lemma 1 presented in Appendix, for given values of* *l*, *L*_*x*_, *L*_*y*_, *M*_*x*_ *and* *e* > 0, *under the null hypothesis that* {*Y*_*t*_} *does not strictly Granger cause* {*X*_*t*_}, *then the test statistic is defined in* (5)

$$\begin{array}{c} \sqrt{n}\left(\frac{{\hat{C}}_{1}\left(M_{x}+L_{x},L_{y},e,l\right)}{{\hat{C}}_{2}\left(L_{x},L_{y},e,l\right)}-\frac{{\hat{C}}_{3}\left(M_{x}+L_{x},e,l\right)}{{\hat{C}}_{4}\left(L_{x},e,l\right)}\right)\overset{d}{⟶}N\left(0,\sigma^{2}\left(M_{x},L_{x},L_{y},e,l\right)\right)\;. \end{array}$$

The asymptotic variance *σ*^2^(*M*_*x*_, *L*_*x*_, *L*_*y*_, *e*, *l*) with its consistent estimator ${\hat{\sigma }}^{2}\left(M_{x},L_{x},L_{y},e,l\right)$ and the proof of theorem 1 are given in the Appendix (With some basic definitions from [17]). The hypothesis *H*_0_ defined in (4) is rejected at *α* if

$$\begin{array}{c} \left|T_{n}\right|/\hat{\sigma }\left(M_{x},L_{x},L_{y},e,l\right)>z_{\alpha /2}, \end{array}$$

where *z*_*α*/2_ is the up *α*/2 quantile of the standard normal distribution. In this situation, we will conclude that there exists nonlinear Granger causality from {*Y*_*t*_} to {*X*_*t*_}.

There are several possible methods to estimate the asymptotic variance *σ*^2^(*M*_*x*_, *L*_*x*_, *L*_*y*_, *e*, *l*). A model-based approach uses known laws of {*X*_*t*_} and {*Y*_*t*_} to calculate the expectations in the formula given in the Appendix and simply substitutes *C*_*j*_(∗), *j* = 1, 2, 3, 4 with their corresponding estimates. However, in practice, we can hardly avoid model misspecification and may obtain improper laws of {*X*_*t*_} and {*Y*_*t*_}. We suggest the use of bootstrap methods as in the simulation studies we use to test hypothesis *H*_0_.

## Numerical study

### Simulaiton

In this subsection, we perform numerical studies using simulations to illustrate the applicability and superiority of the new multivariate nonlinear Granger causality test developed in Section 3. Let *R* be the times of rejecting the null hypothesis that *Y*_*t*_ does not strictly Granger cause *X*_*t*_ nonlinearly in 10,000 replications at the *α* level, and thus, the empirical power is *R*/10,000. In our simulation, the level *α* = 0.05, we standardized the series and chose the same lag length and lead length: *L*_*x*_ = *L*_*y*_ = *M*_*x*_ = 1. We set three situations of *l* and two situations of *e*: *l* = 1, *l* = 2, *l* = 3 and *e* = 1, *e* = 1.5.

Consider the following model:

$$\begin{array}{c} X_{t}=\beta Y_{1,t-1}Y_{2,t-1}+\varepsilon_{t}\;, \end{array}$$ (6)

where {(*Y*_1,*t*_, *Y*_2,*t*_)′} are i.i.d. and mutually independent random variables generated from standard normal distribution *N*(0, 1), {*ε*_*t*_} is Gaussian white noise generated from *N*(0, 0.1) and independent of {*Y*_1,*t*_}, {*Y*_2,*t*_}. There is no nonlinear Granger causality from *Y*_*t*_ to *X*_*t*_ when *β* = 0, and causality strengthens when *β* increases.

From the results displayed in Table 2, we conclude first that our test possesses decent size, as we can see when *β* = 0 the empirical size are all closed to the test level 0.05 for different settings of parameters and sample size. Second, our test possesses very appropriate power, as we see that empirical power increases as *β* increases, especially when sample size is 500 the empirical power sharply increase to 1. Further, we find that different settings of *e* may influence the test results. Though the influence is little in our simulation, we still suggest that practitioners choose a couple of different values of *e*.

**Table 2:** Test multivariate nonlinear Granger causality form *Y*_*t*_ to *X*_*t*_.

*T* = 200	*e* = 1			*e* = 1.5		
	*l* = 1	*l* = 2	*l* = 3	*l* = 1	*l* = 2	*l* = 3
*β* = 0	0.0419	0.0441	0.0432	0.0444	0.0506	0.0438
*β* = 0.1	0.1509	0.2341	0.2184	0.3647	0.5209	0.5121
*β* = 0.2	0.5629	0.7425	0.7284	0.8352	0.9416	0.9416
*β* = 0.3	0.8178	0.9323	0.9267	0.929	0.9810	0.9825
*β* = 0.4	0.8994	0.9712	0.9719	0.9512	0.9878	0.9889
*β* = 0.5	0.9366	0.9812	0.9808	0.9586	0.9914	0.9915
*β* = 0.6	0.9475	0.9870	0.9862	0.9640	0.9918	0.9940
*β* = 0.7	0.9574	0.9875	0.9874	0.9664	0.9921	0.9942
*β* = 0.8	0.9615	0.9888	0.9882	0.9688	0.9926	0.9953
*β* = 0.9	0.9633	0.9896	0.9901	0.9711	0.9923	0.994
*T* = 500	*e* = 1			*e* = 1.5		
	*l* = 1	*l* = 2	*l* = 3	*l* = 1	*l* = 2	*l* = 3
*β* = 0	0.0560	0.0560	0.0478	0.0501	0.0529	0.0423
*β* = 0.1	0.3969	0.5081	0.5344	0.7835	0.9043	0.9017
*β* = 0.2	0.9622	0.9908	0.9900	0.9994	1.0000	1.0000
*β* = 0.3	0.9989	1.0000	0.9997	0.9999	1.0000	1.0000
*β* = 0.4	0.9998	1.0000	1.0000	1.0000	1.0000	1.0000
*β* = 0.5	1.0000	1.0000	1.0000	1.0000	1.0000	1.0000
*β* = 0.6	1.0000	1.0000	1.0000	1.0000	1.0000	1.0000
*β* = 0.7	1.0000	1.0000	1.0000	1.0000	1.0000	1.0000
*β* = 0.8	1.0000	1.0000	1.0000	1.0000	1.0000	1.0000
*β* = 0.9	1.0000	1.0000	1.0000	1.0000	1.0000	1.0000

Note: *Y*_*t*_ and *X*_*t*_ are from the model present in Eq (6). *L*_*x*_ = *L*_*y*_ = *M*_*x*_ = 1 in our test. Simulation is conducted with the test level *α* = 5%, and 10,000 replications.

### Application

Now we apply our new method to detect the nonlinear causality from returns to exchanging volumes on China’s stock market. To eliminate the potential influence of asymmetry information caused by language barriers, different accounting standards and foreign currencies exchanging, we consider China’s A shares which are denominated in the local currency the Yuan (also called RMB) and traded among China’s citizens. A shares listed on the Shanghai Stock Exchange are named SHA while the ones listed on the Shenzhen Stock Exchange are named SZA.

We denote the prices of SHA and SZA at *t* as $P_{t}^{SH}$ and $P_{t}^{SZ}$ and denote the according trading volumes as $V_{t}^{SH}$ and $V_{t}^{SZ}$. The daily stock returns of SHA and SZA (from Jan. 1, 2010 to Dec. 30, 2016) are expressed as $Y_{1t}=\ln\left(P_{t}^{SH}/P_{t-1}^{SH}\right)$ and $Y_{2t}=\ln\left(P_{t}^{SZ}/P_{t-1}^{SZ}\right)$ respectively, the percentage changes in trading volume are expressed as $X_{1t}=\ln\left(V_{t}^{SH}/V_{t-1}^{SH}\right)$ and $X_{2t}=\ln\left(V_{t}^{SZ}/V_{t-1}^{SZ}\right)$. We apply our new multivariate causality test proposed in section 2.2 to detect the causalities between (*Y*_1*t*_, *Y*_2*t*_)′ and (*X*_1*t*_, *X*_2*t*_)′, we let *L*_*x*_1__ = *L*_*x*_2__ = *L*_*y*_1__ = *L*_*y*_2__ = *m*_*x*_1__ = *m*_*x*_2__ = 1 and consider *l* = 1, *l* = 2 and *l* = 3, *e* = 1 and *e* = 1.5.

Table 3 presents some descriptive statistics of *Y*_1*t*_, *Y*_2*t*_, *X*_1*t*_ and *X*_2*t*_. It is worth noting that the standard deviations of *Y*_1*t*_, *Y*_2*t*_, *X*_1*t*_ and *X*_2*t*_ are 0.014843, 0.017552, 0.195853 and 0.171018 respectively. To implement our proposed test, *Y*_1*t*_, *Y*_2*t*_, *X*_1*t*_ and *X*_2*t*_ need to be standardized at first so that all series share a common standard deviation 1. To avoid tedious notation we also denote the standardized sequences (*Y*_1*t*_ − *Mean*(*Y*_1*t*_))/*SD*(*Y*_1*t*_), (*Y*_2*t*_ − *Mean*(*Y*_2*t*_))/*SD*(*Y*_2*t*_), (*X*_1*t*_ − *Mean*(*X*_1*t*_))/*SD*(*X*_1*t*_) and (*X*_2*t*_ − *Mean*(*X*_2*t*_))/*SD*(*X*_2*t*_) as *Y*_1*t*_, *Y*_2*t*_, *X*_1*t*_ and *X*_2*t*_. Table 4 reports the results of detecting causality from (*Y*_1*t*_, *Y*_2*t*_)′ to (*X*_1*t*_, *X*_2*t*_)′ as well as two pairs of subset, *Y*_1*t*_ to *X*_1*t*_ and *Y*_2*t*_ to *X*_2*t*_. Generally speaking, there exists causality from stock returns to volume changes, as we can see on the row which testing no causality from (*Y*_1*t*_, *Y*_2*t*_)′ → (*X*_1*t*_, *X*_2*t*_)′, *p* values are all much smaller than level 0.05. Further, we try to dig which subset possess the dynamic causal explanatory ability. Results on the last row of Table 3 strongly suggest that the returns of SZA nonlinearly cause the trading volume of SZA. However, the causal explanatory ability is weak from the returns of SHA to the trading volume of SHA. We think these results coincide with practical situation, noticing that companies listed on the Shanghai Stock Exchange are likely to be big state-owned companies, while those listed on the Shenzhen Stock Exchange tend to be smaller export-oriented companies. Investors who invest on big state-owned companies are tend to be value investment and would like to hold their stocks with in a long time but pay less attention on the price changing comparing to these invest on Shenzhen A shares.

**Table 3:** Descriptive statistics of the series *Y*_1*t*_, *Y*_2*t*_, *X*_1*t*_ and *X*_2*t*_.

Series	Min	Max	Mean	Median	Standard Deviation
*Y*_1*t*_	-0.088694	0.055990	-0.000027	0.000482	0.014843
*Y*_2*t*_	-0.086205	0.063390	0.000290	0.001650	0.017552
*X*_1*t*_	-1.219501	1.400890	0.000118	-0.010721	0.195853
*X*_2*t*_	-1.263281	1.380028	0.000490	-0.004409	0.171018

Note: It is worth noting that the standard deviations of the four series are not the same. To implement the test, all series need to be standardized at first so that all series share a common standard deviation 1.

**Table 4:** Testing nonlinear causality from prices to trading volumes of China’s A shares.

*H*_0_: No causality from left to right	*e* = 1			*e* = 1.5		
	*l* = 1	*l* = 2	*l* = 3	*l* = 1	*l* = 2	*l* = 3
(*Y*_1*t*_, *Y*_2*t*_)′ → (*X*_1*t*_, *X*_2*t*_)′	0.0209*	0.0722	0.0096**	0.0077**	0.0089**	0.0013**
*Y*_1*t*_ → *X*_1*t*_	0.0548	0.0296*	0.0251*	0.0052**	0.0609	0.0977
*Y*_2*t*_ → *X*_2*t*_	0.0065**	0.0020**	0.0005**	0.0007**	0.0005**	0.0001**

Note: “∗” and “∗∗” mean significant at level 0.05 and 0.01 respectively.

## Conclusion and remarks

In this paper, we reinvestigate the multivariate nonlinear Granger causality test extended by Bai et al. [9] which attempt to uncover significant nonlinearities in the dynamic interrelationships between two groups of variables. We find that Bai et al. [9] as well as Hiemstra and Jones [8] take the estimators of the probabilities in their definition as *U*-statistics and establish a CLT of the test statistic by applying the asymptotic property of *U*-statistics. After revealing that the estimators proposed by Bai et al. [9] is not *U*- statistics, we show that their estimators are also not consistent.

The procedure of our new test begins with presenting consistent estimators of probabilities in the definition. Numerical study supports that our estimators are consistent, further our new test possesses admirable properties both in size and power.

There are still amounts of appealing aspects in nonlinear Granger causality test. It is worth noting that Diks and Wolski [18] extend the test in Diks and Panchenko [15] which highlight a need for substitutions for the relationship tested in the HJ test.

## Appendix

### A1: Central limit theorems for strong mixing stationary sequence

$\left\{\left(Z_{t},F_{t}\right),-\infty <t<\infty \right\}$ is a stochastic process defined on the probability space (Ω,F,P). The history and the future of *Z*_*t*_ are *σ*-algebras $M_{t}^{\infty }=\left\{F_{s},s>t\right\}$ and *σ*-algebras $M_{-\infty }^{t}=\left\{F_{s},s<t\right\}$ respectively.

Let {$\left(Z_{i},F_{i}\right)$} be a stationary sequence with *E*(*Z*_*i*_) = 0, $E({Z_{i}}^{2})<0$, and set $S_{n}^{m}=\sum_{i=m+1}^{n+m}Z_{i}$, ${\sigma_{n}}^{2}=Var\left(S_{n}^{m}\right)$. We shall say that the sequence satisfies the central limit theorem if

$$\begin{array}{c} \lim_{n\to \infty }P\left\{\frac{S_{n}^{m}}{\sigma_{n}}<z\right\}={\left(2\pi \right)}^{-\frac{1}{2}}\int_{-\infty }^{z}e^{-\frac{1}{2}u^{2}}du=\Phi \left(z\right)\;. \end{array}$$

**Definition A1:** A stationary process {*Z*_*t*_} is said to be strongly mixing (completely regular) if $\alpha \left(\tau \right)=\underset{A\in m_{-\infty }^{0},B\in m_{\tau }^{\infty }}{\sup}\left|P\left(AB\right)-P\left(A\right)P\left(B\right)\right|\to 0$ as *τ* → ∞ through positive values.

**Lemma A1**: Let the stationary sequence {*Z*_*i*_} satisfy the strong mixing condition with mixing coefficient *α*(*n*), and let *E*|*Z*_*i*_|^2+*δ*^ < ∞ for some *δ* > 0. If $\sum_{n=1}^{\infty }{\alpha (n)}^{\delta /(2+\delta )}<\infty$, then $\sigma^{2}=E\left({Z_{0}}^{2}\right)+2\sum_{j=1}^{\infty }E\left(Z_{0}Z_{j}\right)<\infty$, and if *σ* ≠ 0, then $\lim_{n\to \infty }P\left\{\sigma^{-1}n^{-\frac{1}{2}}\sum_{i=1}^{n}Z_{i}<z\right\}=\Phi \left(z\right)$.

Readers can be referred to Ibragimov [17] for a proof and detailed discussion.

### A2: Proof of Theorem 1

Assume {*x*_*i*,1_, *x*_*i*,2_, ⋯, *x*_*i*,*T*_} and {*y*_*j*,1_, *y*_*j*,2_, ⋯, *y*_*j*,*T*_}, *i* ∈ {1, 2, ⋯, *n*_1_}, *j* ∈ {1, 2, ⋯, *n*_2_} are both strong mixing stationary sequences whose mixing coefficient satisfying the conditions in Lemma 1. Then the following four sequences

$$\begin{array}{ccc} & & \{Z_{1t}=n^{-1}\left(\prod_{i=1}^{n_{1}}I\left(x_{i,t-L_{x_{i}}}^{m_{x_{i}}+L_{x_{i}}},x_{i,t+l-L_{x_{i}}}^{m_{x_{i}}+L_{x_{i}}},e\right)\cdot \prod_{j=1}^{n_{2}}I\left(y_{j,t-L_{y_{j}}}^{L_{y_{j}}},y_{j,t+l-L_{y_{j}}}^{L_{y_{j}}},e\right)-C_{1}\left(M_{x}+L_{x},L_{y},e;l\right)\right)\}\;,\\ & & \{Z_{2t}=n^{-1}\left(\prod_{i=1}^{n_{1}}I\left(x_{i,t-L_{x_{i}}}^{L_{x_{i}}},x_{i,t+l-L_{x_{i}}}^{L_{x_{i}}},e\right)\cdot \prod_{j=1}^{n_{2}}I\left(y_{j,t-L_{y_{j}}}^{L_{y_{j}}},y_{j,t+l-L_{y_{j}}}^{L_{y_{j}}},e\right)-C_{2}\left(L_{x},L_{y},e;l\right)\right)\}\;,\\ & & \{Z_{3t}=n^{-1}\left(\prod_{i=1}^{n_{1}}I\left(x_{i,t-L_{x_{i}}}^{m_{x_{i}}+L_{x_{i}}},x_{i,t+l-L_{x_{i}}}^{m_{x_{i}}+L_{x_{i}}},e\right)-C_{3}\left(L_{x},L_{y},e;l\right)\right)\}\;,\\ & & \{Z_{4t}=n^{-1}\left(\prod_{i=1}^{n_{1}}I\left(x_{i,t-L_{x_{i}}}^{L_{x_{i}}},x_{i,t+l-L_{x_{i}}}^{L_{x_{i}}},e\right)-C_{4}\left(L_{x},e;l\right)\right)\}\;,\\ & & t=L_{xy}+1,\cdots ,T-l-m_{x}+1, \end{array}$$

satisfy the conditions of Lemma 1, where *n* = *T* − *L*_*xy*_ − *l* − *m*_*x*_ + 1 and

$$\begin{array}{ccc} & & C_{1}\left(M_{x}+L_{x},L_{y},e;l\right)\equiv P\left(∥X_{t-L_{x}}^{M_{x}+L_{x}}-X_{t+l-L_{x}}^{M_{x}+L_{x}}∥<e,∥Y_{t-L_{y}}^{L_{y}}-Y_{t+l-L_{y}}^{L_{y}}∥<e\right)\;,\\ & & C_{2}\left(L_{x},L_{y},e;l\right)\equiv P\left(∥X_{t-L_{x}}^{L_{x}}-X_{t+l-L_{x}}^{L_{x}}∥<e,∥Y_{t-L_{y}}^{L_{y}}-Y_{t+l-L_{y}}^{L_{y}}∥<e\right)\\ & & C_{3}\left(M_{x}+L_{x},e;l\right)\equiv P\left(∥X_{t-L_{x}}^{M_{x}+L_{x}}-X_{t+l-L_{x}}^{M_{x}+L_{x}}∥<e\right)\\ & & C_{4}\left(L_{x},e;l\right)\equiv P\left(∥X_{t-L_{x}}^{L_{x}}-X_{t+l-L_{x}}^{L_{x}}∥<e\right)\;. \end{array}$$

So {*Z*_1*t*_}, {*Z*_2*t*_}, {*Z*_3*t*_} and {*Z*_4*t*_} satisfy the central limit theorem.

Further, for any real number *a*_1_, *a*_2_, *a*_3_ and *a*_4_, the sequence {*Z*_*t*_ = *a*_1_ *Z*_1*t*_ + *a*_2_ *Z*_2*t*_ + *a*_3_ *Z*_3*t*_ + *a*_4_ *Z*_4*t*_, *t* = *L*_*xy*_ + 1, ⋯, *T* − *l* − *L*_*xy*_ − *m*_*x*_ + 1} also satisfies the conditions of Lemma 1 which implying that

$$\begin{array}{c} \sqrt{n}\left[\begin{array}{c} {\hat{C}}_{1}\left(M_{x}+L_{x},L_{y},e;l\right)-C_{1}\left(M_{x}+L_{x},L_{y},e;l\right)\\ {\hat{C}}_{2}\left(L_{x},L_{y},e;l\right)-C_{2}\left(L_{x},L_{y},e;l\right)\\ {\hat{C}}_{3}\left(M_{x}+L_{x},e;l\right)-C_{3}\left(M_{x}+L_{x},e;l\right)\\ {\hat{C}}_{4}\left(L_{x},e;l\right)-C_{4}\left(L_{x},e;l\right) \end{array}\right]\overset{d}{⟶}N\left(0,\Sigma \right), \end{array}$$

where

$$\begin{array}{ccc} & & {\hat{C}}_{1}\left(M_{x}+L_{x},L_{y},e;l\right)\equiv \frac{1}{n}\sum_{t=L_{xy}+1}^{T-l-m_{x}+1}\prod_{i=1}^{n_{1}}I\left(x_{i,t-L_{x_{i}}}^{m_{x_{i}}+L_{x_{i}}},x_{i,t+l-L_{x_{i}}}^{m_{x_{i}}+L_{x_{i}}},e\right)\cdot \prod_{j=1}^{n_{2}}I\left(y_{j,t-L_{y_{j}}}^{L_{y_{j}}},y_{j,t+l-L_{y_{j}}}^{L_{y_{j}}},e\right)\\ & & {\hat{C}}_{2}\left(L_{x},L_{y},e;l\right)\equiv \frac{1}{n}\sum_{t=L_{xy}+1}^{T-l-m_{x}+1}\prod_{i=1}^{n_{1}}I\left(x_{i,t-L_{x_{i}}}^{L_{x_{i}}},x_{i,t+l-L_{x_{i}}}^{L_{x_{i}}},e\right)\cdot \prod_{j=1}^{n_{2}}I\left(y_{j,t-L_{y_{j}}}^{L_{y_{j}}},y_{j,t+l-L_{y_{j}}}^{L_{y_{j}}},e\right)\\ & & {\hat{C}}_{3}\left(M_{x}+L_{y},e;l\right)\equiv \frac{1}{n}\sum_{t=L_{xy}+1}^{T-l-m_{x}+1}\prod_{i=1}^{n_{1}}I\left(x_{i,t-L_{x_{i}}}^{m_{x_{i}}+L_{x_{i}}},x_{i,t+l-L_{x_{i}}}^{m_{x_{i}}+L_{x_{i}}},e\right)\\ & & {\hat{C}}_{4}\left(L_{x},e;l\right)\equiv \frac{1}{n}\sum_{t=L_{xy}+1}^{T-l-m_{x}+1}\prod_{i=1}^{n_{1}}I\left(x_{i,t-L_{x_{i}}}^{L_{x_{i}}},x_{i,t+l-L_{x_{i}}}^{L_{x_{i}}},e\right) \end{array}$$

and **Σ** is a 4 × 4 symmetric matrix. Denote

$$\begin{array}{c} h_{1}\left(L_{x},L_{y},M_{x},l,k\right)=I\left(x_{L_{xy}+1+k-L_{x}}^{L_{x}+M_{x}},x_{L_{xy}+1+k+l-L_{x}}^{L_{x}+M_{x}},e\right)\;, \end{array}$$

$$\begin{array}{c} h_{2}\left(L_{x},L_{y},l,k\right)=I\left(y_{L_{xy}+1+k-L_{y}}^{L_{y}},y_{L_{xy}+1+k+l-L_{y}}^{L_{y}},e\right)\;, \end{array}$$

We have

$$\begin{array}{ccc} \Sigma_{11} & = & E[{\left(h_{1}\left(L_{x},L_{y},M_{x},l,0\right)h_{2}\left(L_{x},L_{y},l,0\right)-C_{1}\left(M_{x}+L_{x},L_{y},e;l\right)\right)}^{2}]\\ & & +\sum_{k=1}^{n-1}2\left(1-\frac{k}{n}\right)E[\left(h_{1}\left(L_{x},L_{y},M_{x},l,0\right)h_{2}\left(L_{x},L_{y},l,0\right)-C_{1}\left(M_{x}+L_{x},L_{y},e;l\right)\right)\\ & & \;(h_{1}\left(L_{x},L_{y},M_{x},l,k\right)h_{2}\left(L_{x},L_{y},l,k\right)-C_{1}\left(M_{x}+L_{x},L_{y},e;l\right))]\;,\\ \Sigma_{12} & = & E[\left(h_{1}\left(L_{x},L_{y},M_{x},l,0\right)h_{2}\left(L_{x},L_{y},l,0\right)-C_{1}\left(M_{x}+L_{x},L_{y},e;l\right)\right)\\ & & \;(h_{1}\left(L_{x},L_{y},0,l,0\right)h_{2}\left(L_{x},L_{y},l,0\right)-C_{2}\left(L_{x},L_{y},e;l\right))]\\ & & +\sum_{k=1}^{n-1}\left(1-\frac{k}{n}\right)E[\left(h_{1}\left(L_{x},L_{y},M_{x},l,0\right)h_{2}\left(L_{x},L_{y},l,0\right)-C_{1}\left(M_{x}+L_{x},L_{y},e;l\right)\right)\\ & & \;(h_{1}\left(L_{x},L_{y},0,l,k\right)h_{2}\left(L_{x},L_{y},l,k\right)-C_{2}\left(L_{x},L_{y},e;l\right))]\\ & & +\sum_{k=1}^{n-1}\left(1-\frac{k}{n}\right)E[\left(h_{1}\left(L_{x},L_{y},M_{x},l,k\right)h_{2}\left(L_{x},L_{y},l,k\right)-C_{1}\left(M_{x}+L_{x},L_{y},e;l\right)\right)\\ & & \;(h_{1}\left(L_{x},L_{y},0,l,0\right)h_{2}\left(L_{x},L_{y},l,0\right)-C_{2}\left(L_{x},L_{y},e;l\right))]\;,\\ \Sigma_{13} & = & E[\left(h_{1}\left(L_{x},L_{y},M_{x},l,0\right)h_{2}\left(L_{x},L_{y},l,0\right)-C_{1}\left(M_{x}+L_{x},L_{y},e;l\right)\right)\\ & & \;(h_{1}\left(L_{x},L_{y},M_{x},l,0\right)-C_{3}\left(M_{x}+L_{x},e;l\right))]\\ & & +\sum_{k=1}^{n-1}\left(1-\frac{k}{n}\right)E[\left(h_{1}\left(L_{x},L_{y},M_{x},l,0\right)h_{2}\left(L_{x},L_{y},l,0\right)-C_{1}\left(M_{x}+L_{x},L_{y},e;l\right)\right)\\ & & \;(h_{1}\left(L_{x},L_{y},M_{x},l,k\right)-C_{3}\left(M_{x}+L_{x},e;l\right))]\\ & & +\sum_{k=1}^{n-1}\left(1-\frac{k}{n}\right)E[\left(h_{1}\left(L_{x},L_{y},M_{x},l,k\right)h_{2}\left(L_{x},L_{y},l,k\right)-C_{1}\left(M_{x}+L_{x},L_{y},e;l\right)\right)\\ & & \;(h_{1}\left(L_{x},L_{y},M_{x},l,0\right)-C_{3}\left(M_{x}+L_{x},e;l\right))]\;, \end{array}$$

$$\begin{array}{ccc} \Sigma_{14} & = & E[\left(h_{1}\left(L_{x},L_{y},M_{x},l,0\right)h_{2}\left(L_{x},L_{y},l,0\right)-C_{1}\left(M_{x}+L_{x},L_{y},e;l\right)\right)\\ & & \;(h_{1}\left(L_{x},L_{y},0,l,0\right)-C_{4}\left(L_{x},e;l\right))]\\ & & +\sum_{k=1}^{n-1}\left(1-\frac{k}{n}\right)E[\left(h_{1}\left(L_{x},L_{y},M_{x},l,0\right)h_{2}\left(L_{x},L_{y},l,0\right)-C_{1}\left(M_{x}+L_{x},L_{y},e;l\right)\right)\\ & & \;(h_{1}\left(L_{x},L_{y},0,l,k\right)-C_{4}\left(L_{x},e;l\right))]\\ & & +\sum_{k=1}^{n-1}\left(1-\frac{k}{n}\right)E[\left(h_{1}\left(L_{x},L_{y},M_{x},l,k\right)h_{2}\left(L_{x},L_{y},l,k\right)-C_{1}\left(M_{x}+L_{x},L_{y},e;l\right)\right)\\ & & \;(h_{1}\left(L_{x},L_{y},0,l,0\right)-C_{4}\left(L_{x},e;l\right))]\;,\\ \Sigma_{22} & = & E[{\left(h_{1}\left(L_{x},L_{y},0,l,0\right)h_{2}\left(L_{x},L_{y},l,0\right)-C_{2}\left(L_{x},L_{y},e;l\right)\right)}^{2}]\\ & & +\sum_{k=1}^{n-1}2\left(1-\frac{k}{n}\right)E[\left(h_{1}\left(L_{x},L_{y},0,l,0\right)h_{2}\left(L_{x},L_{y},l,0\right)-C_{2}\left(L_{x},L_{y},e;l\right)\right)\\ & & \;(h_{1}\left(L_{x},L_{y},0,l,k\right)h_{2}\left(L_{x},L_{y},l,k\right)-C_{2}\left(L_{x},L_{y},e;l\right))]\;,\\ \Sigma_{23} & = & E[\left(h_{1}\left(L_{x},L_{y},0,l,0\right)h_{2}\left(L_{x},L_{y},l,0\right)-C_{2}\left(L_{x},L_{y},e;l\right)\right)\\ & & \;(h_{1}\left(L_{x},L_{y},M_{x},l,0\right)-C_{3}\left(M_{x}+L_{x},e;l\right))]\\ & & +\sum_{k=1}^{n-1}\left(1-\frac{k}{n}\right)E[\left(h_{1}\left(L_{x},L_{y},0,l,0\right)h_{2}\left(L_{x},L_{y},l,0\right)-C_{2}\left(L_{x},L_{y},e;l\right)\right)\\ & & \;(h_{1}\left(L_{x},L_{y},M_{x},l,k\right)-C_{3}\left(M_{x}+L_{x},e;l\right))]\\ & & +\sum_{k=1}^{n-1}\left(1-\frac{k}{n}\right)E[\left(h_{1}\left(L_{x},L_{y},0,l,k\right)h_{2}\left(L_{x},L_{y},l,k\right)-C_{2}\left(L_{x},L_{y},e;l\right)\right)\\ & & \;(h_{1}\left(L_{x},L_{y},M_{x},l,0\right)-C_{3}\left(M_{x}+L_{x},e;l\right))]\;, \end{array}$$

$$\begin{array}{ccc} \Sigma_{24} & = & E[\left(h_{1}\left(L_{x},L_{y},0,l,0\right)h_{2}\left(L_{x},L_{y},l,0\right)-C_{2}\left(L_{x},L_{y},e;l\right)\right)\\ & & \;(h_{1}\left(L_{x},L_{y},0,l,0\right)-C_{4}\left(L_{x},e;l\right))]\\ & & +\sum_{k=1}^{n-1}\left(1-\frac{k}{n}\right)E[\left(h_{1}\left(L_{x},L_{y},0,l,0\right)h_{2}\left(L_{x},L_{y},l,0\right)-C_{2}\left(L_{x},L_{y},e;l\right)\right)\\ & & \;(h_{1}\left(L_{x},L_{y},0,l,k\right)-C_{4}\left(L_{x},e;l\right))]\\ & & +\sum_{k=1}^{n-1}\left(1-\frac{k}{n}\right)E[\left(h_{1}\left(L_{x},L_{y},0,l,k\right)h_{2}\left(L_{x},L_{y},l,k\right)-C_{2}\left(L_{x},L_{y},e;l\right)\right)\\ & & \;(h_{1}\left(L_{x},L_{y},0,l,0\right)-C_{4}\left(L_{x},e;l\right))]\;,\\ \Sigma_{33} & = & E[{\left(h_{1}\left(L_{x},L_{y},M_{x},l,0\right)-C_{3}\left(M_{x}+L_{x},e;l\right)\right)}^{2}]\\ & & +\sum_{k=1}^{n-1}2\left(1-\frac{k}{n}\right)E[\left(h_{1}\left(L_{x},L_{y},M_{x},l,0\right)-C_{3}\left(M_{x}+L_{x},e;l\right)\right)\\ & & \;(h_{1}\left(L_{x},L_{y},M_{x},l,k\right)-C_{3}\left(M_{x}+L_{x},e;l\right))]\;,\\ \Sigma_{34} & = & E[\left(h_{1}\left(L_{x},L_{y},M_{x},l,0\right)-C_{3}\left(M_{x}+L_{x},e;l\right)\right)\\ & & \;(h_{1}\left(L_{x},L_{y},0,l,0\right)-C_{4}\left(L_{x},e;l\right))]\\ & & +\sum_{k=1}^{n-1}\left(1-\frac{k}{n}\right)E[\left(h_{1}\left(L_{x},L_{y},M_{x},l,0\right)-C_{3}\left(M_{x}+L_{x},e;l\right)\right)\\ & & \;(h_{1}\left(L_{x},L_{y},0,l,k\right)-C_{4}\left(L_{x},e;l\right))]\\ & & +\sum_{k=1}^{n-1}\left(1-\frac{k}{n}\right)E[\left(h_{1}\left(L_{x},L_{y},M_{x},l,k\right)-C_{3}\left(M_{x}+L_{x},e;l\right)\right)\\ & & \;(h_{1}\left(L_{x},L_{y},0,l,0\right)-C_{4}\left(L_{x},e;l\right))]\;,\\ \Sigma_{44} & = & E[{\left(h_{1}\left(L_{x},L_{y},0,l,0\right)-C_{4}\left(L_{x},e;l\right)\right)}^{2}]\\ & & +\sum_{k=1}^{n-1}2\left(1-\frac{k}{n}\right)E[\left(h_{1}\left(L_{x},L_{y},0,l,0\right)-C_{4}\left(L_{x},e;l\right)\right)\\ & & \;(h_{1}\left(L_{x},L_{y},0,l,k\right)-C_{4}\left(L_{x},e;l\right))]\;. \end{array}$$

Under the null hypothesis, applying the delta method (Serfling, 1980), we have

$$\begin{array}{c} \sqrt{n}\left(\frac{{\hat{C}}_{1}\left(M_{x}+L_{x},L_{y},e,l\right)}{{\hat{C}}_{2}\left(L_{x},L_{y},e,l\right)}-\frac{{\hat{C}}_{3}\left(M_{x}+L_{x},e,l\right)}{{\hat{C}}_{4}\left(L_{x},e,l\right)}\right)\overset{d}{⟶}N\left(0,\sigma^{2}\left(M_{x},L_{x},L_{y},e,l\right)\right)\;, \end{array}$$

where *σ*^2^(*M*_*x*_, *L*_*x*_, *L*_*y*_, *e*, *l*) = ∇′ **Σ**∇, in which

$$\begin{array}{ccc} \nabla & = & {\left(\frac{1}{C_{2}\left(L_{x},L_{y},e,l\right)}\;,\;-\frac{C_{1}\left(M_{x}+L_{x},L_{y},e,l\right)}{C_{2}^{2}\left(L_{x},L_{y},e,l\right)}\;,\;-\frac{1}{C_{4}\left(L_{x},e,l\right)}\;,\;\frac{C_{3}\left(M_{x}+L_{x},e,l\right)}{C_{4}^{2}\left(L_{x},e,l\right)}\right)}^{\prime }\;. \end{array}$$

An consistent estimator ${\hat{\sigma }}^{2}\left(M_{x},L_{x},L_{y},e,l\right)$ of the asymptotic variance can be got by replacing all the parts in the sandwich ∇′ **Σ**∇ by their empirical estimates.
